# Factors Associated with Increased Healthcare Utilization Within 30 and 90 Days Post-Discharge of CAR-T Cell Therapy in Patients with R/R LBCL

**DOI:** 10.3390/hematolrep18040052

**Published:** 2026-07-27

**Authors:** Philip Yeung, Aaron Trando, Ah-Reum Jeong, Dimitrios Tzachanis

**Affiliations:** 1 Master of Advanced Studies (MAS) Program in Clinical Research, University of California San Diego, La Jolla, CA 92093, USA; 2 KUMC Master of Science Health Data Science Program, University of Kansas Medical Center, Kansas City, KS 66160, USA; 3 Department of Internal Medicine, University of California San Diego, La Jolla, CA 92093, USA; 4 Department of Medicine, Division of Blood and Marrow Transplantation, University of California San Diego, La Jolla, CA 92093, USA; 5 Division of Hematology/Oncology, Scripps Cancer Center, La Jolla, CA 92037, USA

**Keywords:** chimeric antigen receptor T-cell therapy, axicabtagene ciloleucel, tisagenlecleucel, large B-cell lymphoma, allogeneic hematopoietic stem cell transplant, hospital readmission, hospital length of stay, healthcare utilization

## Abstract

**Background/Objectives**: CAR T-cell therapy is a highly efficacious therapy option for relapsed/refractory (R/R) large B-cell lymphoma (LBCL), but its widespread use is currently limited by safety concerns and high healthcare costs. **Methods**: We evaluated 66 patients with R/R LBCL treated with tisagenlecleucel or axicabtagene ciloleucel between 2016 and 2022 at a single institution to identify factors associated with increased healthcare utilization. **Results**: The median age of our cohort was 59.5 years, with 22.7% over the age of 70. The median length of stay (LOS) during the initial hospitalization was 12 days (range, 7–62 days). Longer initial hospital LOS was linked to higher age-adjusted HCT-CI score (IRR 1.08; 95% CI, 1.01 to 1.15; *p* = 0.0203), thrombocytopenia grade 3–4 (IRR 1.32; 95% CI, 1.07 to 1.63; *p* = 0.0091), and first ICU admission (IRR 1.81; 95% CI, 1.37 to 2.38; *p* < 0.00001). Thirty- and 90-day readmission rates post-discharge after receiving CAR T-cell therapy were 21.2% and 28.8%, respectively. Multiple readmissions within 90 days after initial hospital discharge were observed in 15% of patients. Longer 30-day readmission LOS was linked to initial hospital LOS (IRR 1.23; 95% CI, 1.11 to 1.35; *p* < 0.0001) but outpatient follow-up was associated with shorter 30-day readmission LOS (IRR 0.43; 95% CI, 0.26–0.69; *p* = 0.0005). Factors associated with longer 90-day readmission LOS included a greater number of ER visits within 60 days of CAR T-cell therapy (IRR 3.10; 95% CI, 2.15–4.46), prior 30-day readmission LOS (IRR 1.35; 95% CI, 1.14–1.60), and SUVmax at Day 30 (IRR 1.06; 95% CI, 1.03–1.09) (all *p*-values < 0.0001). **Conclusions**: Our findings identify key clinical and utilization variables associated with initial and repeat hospitalizations post-CAR T-cell therapy, emphasizing the need for targeted interventions to reduce readmissions and optimize post-discharge care.

## 1. Introduction

CAR T-cell therapy represents a major advancement in the treatment of relapsed/refractory large B-cell lymphoma (R/R LBCL), but its clinical access and utilization is often limited by toxicity and post-treatment complications. Several CD19-directed CAR-T products, including axicabtagene ciloleucel (axi-cel), tisagenlecleucel (tisa-cel), and lisocabtagene maraleucel, have demonstrated durable responses in both clinical trial and real-world settings for R/R LBCL [1,2,3].

Toxicities such as cytokine release syndrome (CRS), immune effector cell-associated neurotoxicity syndrome (ICANS), and infections are well-established adverse effects that frequently lead to intensive care unit (ICU) admission or rehospitalization [4,5,6,7,8,9,10]. Large observational studies have evaluated real-world healthcare utilization data among CAR T-cell recipients, especially among older adults and Medicare Fee-for-Service beneficiaries [11,12]. These analyses have shown that most patients receive CAR T-cell therapy in the inpatient setting, although rates of outpatient administration are increasing, which may reduce overall costs. Nonetheless, 30-day readmission rates remain high, consistently reported between 20% and 30% [9,13,14]. Importantly, these prior studies rely largely on claims or registry data, which often lack clinical granularity, limiting their ability to assess biologic and treatment-related predictors of adverse outcomes. Moreover, the role of post-discharge outpatient care in modifying these outcomes remains poorly understood. Addressing these gaps requires the integration of detailed clinical data, such as laboratory markers, imaging results, and outpatient visit history.

To bridge this evidence gap, we conducted a retrospective cohort study at a single academic institution to evaluate (1) factors associated with longer initial hospital LOS, (2) factors associated with 30- and 90-day readmissions after discharge, and (3) patterns of multiple readmissions within 90 days.

## 2. Materials and Methods

This retrospective study included patients with relapsed/refractory diffuse large B-cell lymphoma (R/R DLBCL) who underwent CAR T-cell therapy. Patients with R/R DLBCL who received either axi-cel or tisa-cel at the University of California of San Diego (UCSD) between January 2016 and June 2022 were included; the date of data cutoff was 15 April 2023. The study was approved by the UCSD Institutional Review Board and adhered to institutional guidelines and the principles of the Declaration of Helsinki.

### 2.1. Data Extraction

Clinical and healthcare utilization data were extracted from electronic medical records to assess hospital LOS, ICU requirements, and readmission outcomes. Extracted variables included baseline demographics, disease characteristics, toxicities, biomarkers, initial hospital LOS, ICU admissions, emergency room (ER) and outpatient (OP) visits, readmissions within 30-, 60-, and 90-days post-discharge, and reasons for readmission. Among disease characteristics, positron emission tomography (PET) data were collected at time of CAR T-cell administration (PET0) and 30 days after CAR T-cell therapy (PET30), per the Deauville scoring criteria; maximum standardized uptake values (SUVmax) were also recorded at the time of the PET30 scan. Initial hospital LOS was defined as the number of days from Day 0 (CAR T-cell administration) to discharge. All readmission endpoints were calculated from the date of discharge after the initial CAR T cell hospitalization. Multiple readmissions were defined as >1 readmission post-discharge within a 90-day period.

### 2.2. Statistical Analysis

Descriptive statistics were used to summarize patient characteristics and other variables. Group comparisons were conducted using chi-square tests, *t*-tests, or non-parametric alternatives depending on distribution. Notably, continuous baseline characteristics and healthcare utilization data were evaluated for normality. Descriptive statistics for continuous variables were generally summarized using medians (min–max) due to the right-skewed nature of these data and presence of outliers, although nonparametric methods may lack power as compared with parametric methods to detect between group differences for a small sample size [15]. However, for zero-inflated variables where the median value was zero (e.g., ICU length of stay, number of ICU admissions), means and standard deviations were selectively reported. Instead of relying solely on non-parametric summaries in zero-inflated variables, which obscures the severity of the clinical course, this approach was chosen to ensure the outputs remained clinically meaningful, accurately reflecting the intensive resource burden driven by the subset of high-acuity patients.

Spearman correlations assessed collinearity among continuous variables [16]. Predictors of readmission were analyzed using univariate and multivariable logistic regression, while hospital LOS outcomes were analyzed using negative binomial regression [17]. These methods were selected based on the nature of the outcome variables: logistic regression for binary outcomes (readmission yes/no) and count-based models for LOS.

With regard to multivariate model generation, to minimize overfitting and preserve model stability in this relatively small cohort (*N* = 66), we implemented a prespecified two-stage variable selection strategy. Candidate predictors were first identified based on clinical relevance and prior evidence. Variables demonstrating potential associations (*p* < 0.20) with the outcome at univariable screenings and clinical variables of interest were subsequently entered into the multivariable count or logistic regression models. Final models were derived using backward stepwise selection with Akaike Information Criterion (AIC) minimization to achieve a parsimonious balance between model fit and complexity. For count outcomes, Poisson and negative binomial models were compared, with the final model selected based on goodness of fit and dispersion characteristics. Robust estimation was used to obtain robust standard errors; model diagnostics included assessment of variance, multicollinearity, and residual fit [16,18]. Consistent with established recommendations for events-per-variable considerations, the final models were limited to a maximum of six predictors to reduce the risk of model overfitting and unstable coefficient estimates. Given the modest sample size and exploratory design, these multivariable analyses should be interpreted as hypothesis-generating and intended to identify clinically relevant associations rather than establish definitive predictive models.

To evaluate the potential influence of overlapping clinical pathways among highly interrelated post-CAR-T healthcare utilization variables (e.g., severe infection, ICU admission, and initial hospital length of stay [LOS]), we assessed multicollinearity using variance inflation factors (VIFs) for all final multivariable models. All VIF values were <5, indicating that problematic multicollinearity was unlikely to have materially affected parameter estimation. Clinically, these variables were considered complementary markers of disease severity and healthcare utilization rather than completely independent biologic processes. Negative binomial models were estimated using robust standard error estimation which was utilized to minimize standard error inflation and to provide valid statistical inference for the final model. Nevertheless, because several predictors represent sequential events occurring along the same clinical trajectory, their estimated associations should be interpreted as adjusted associations rather than evidence of independent causal effects.

To evaluate longer-term healthcare utilization while minimizing temporal overlap between predictors and outcomes, we implemented a Day 30 landmark analysis for the 90-day readmission models. Specifically, readmission length of stay during the first 30 days following discharge was evaluated as an antecedent predictor, whereas the dependent outcome was redefined as readmission length of stay occurring exclusively between Days 31 and 90. This restructuring ensured that the primary predictor and outcome windows were temporally distinct, thereby avoiding part–whole overlap in the primary analysis. In addition, the cumulative number of emergency department visits through Day 60 was included as a marker of evolving post-discharge healthcare utilization. Accordingly, this variable should be interpreted as a time-updated utilization measure rather than a strictly baseline predictor.

As an exploratory measure, overall survival (OS) was estimated using the Kaplan–Meier method and compared between patients with and without at least one unplanned readmission within 90 days following CAR T-cell infusion. Because visual inspection suggested crossing survival curves, both the conventional log-rank test and a Fleming–Harrington weighted log-rank test (ρ = 1, γ = 0) were performed to evaluate potential differences in survival while accounting for non-proportional survival patterns.

A 2-sided *p* < 0.05 was considered as statistically significant. All statistical analyses were performed using R studio version 2025.06.0., with this package including GTsummary, Hmisc, MASS, Sandwich, and Tidyverse.

During the preparation of this manuscript/study, the authors used Google Gemini 3.0 for the purpose of generating figures and checking grammatical errors. The authors have reviewed and edited the output and take full responsibility for the content of this publication.

## 3. Results

### 3.1. Baseline Patient Characteristics

Among the 66 patients included, the median age was 59.5 years (range, 23–81). Fifteen patients (22.7%) within the cohort were over the age of 70, and 67% were male. Axi-cel was administered to 89% of patients, and tisa-cel to 11%. All patients received CAR T-cell therapy in the inpatient setting. Other patient characteristics were discussed in detail in our previous report [19].

The characteristics of patients who experienced 30-/90-day readmissions after initial hospitalization discharge are described in Table 1. The 30- and 90-day readmission rates were 21.2% and 28.8%, respectively. For both 30-/90-day readmission, baseline patient characteristics were similar between non-readmitted and readmitted patients. The proportion of patients aged ≥65 and ≥70 were generally similar across readmission categories, though there was a slight increase in those ≥70 among readmitted patients (especially within the 90-day readmission cohort: 31.6% vs. 19.1%; Fisher’s exact test, *p* = 0.57). ECOG score, the presence of extranodal involvement, and the proportion of double expressors (MYC and BCL2/6) and double-hit lymphoma cases were also comparable across readmission groups.

Upon assessing previous treatment history, patients had a similar number of prior therapies (median 3, range 1–7). The proportion of patients who received bridging therapy did not differ significantly between readmitted and non-readmitted patients. Autologous transplant history was more frequent among readmitted patients, particularly at 30 days (42.9% vs. 25.0%; Fisher’s exact test, *p* = 0.19).

No significant differences in comorbidity index by age-adjusted HCT-CI scores were noted. Median baseline C-reactive protein (CRP) levels were comparable between readmitted and non-readmitted patients at 30 days (1.37 mg/dL vs. 1.23 mg/dL) and at 90 days (1.11 mg/dL vs. 1.38 mg/dL). Median baseline ferritin levels were elevated in readmitted patients at both timepoints (e.g., 1759 ng/mL vs. 776 ng/mL at 30 days and 1372.5 ng/mL vs. 771 ng/mL at 90 days) but were not statistically significant (Wilcoxon rank test, *p* = 0.181 and *p* = 0.163, respectively).

For PET scan metrics, baseline PET0 Deauville scores were similar among readmitted and non-readmitted patients by both 30 and 90 days. Forty-nine patients had PET30 data available (PET30 scans were captured at any time point reasonably around 1 month after CAR T-cell infusion) with SUVmax data available for 44 of these patients. Within the 90-day readmission cohort, median Day30 maximum standardized uptake values (SUVmaxD30) were numerically higher in readmitted patients (9.4 vs. 3.35; Wilcoxon rank test, *p* = 0.0754).

Among 15 readmissions within 30 days of CAR T-cell therapy discharge, 3 were due to infection (including neutropenic fever, *n* = 2, and biliary sepsis, *n* = 1), 2 due to disease progression, 2 due to CRS, and 4 due to ICANS (2 of which had concurrent CRS). Four patients were readmitted due to other reasons, including workup of shortness of breath (*n* = 1), malignant small bowel obstruction (*n* = 1), workup of diaphoresis/nausea/vomiting/hypothermia (*n* = 1), and cardiogenic shock (*n* = 1) (Table 2).

Among 37 readmissions within 90 days of CAR T-cell therapy discharge, 14 were due to infection (including neutropenic fever, *n* = 4; COVID-19 pneumonia, *n* = 3; biliary sepsis, *n* = 2; hemorrhagic cystitis secondary to adenovirus, *n* = 1; clostridium bacteremia, *n* = 1; klebsiella bacteremia, *n* = 1; lower extremity cellulitis, *n* = 1; septic arthritis, *n* = 1), 7 due to disease progression, 2 due to CRS, and 4 due to ICANS. Ten patients were readmitted due to other reasons, including fever of multifactorial etiology (possible IVIG infusion reaction, progressive disease, and infection) (*n* = 1), steroid-induced hyperglycemia (*n* = 1), hypercalcemia (*n* = 1), tumor lysis syndrome (*n* = 1), NSTEMI (*n* = 1), and workup of nasal pain (*n* = 1) (Table 2). There were no further readmissions due to CRS or ICANS post-30 days.

Among the 19 patients readmitted within 90 days of discharge, 14 (73.7%) had at least one readmission within 30 days, 10 (52.6%) between 31 and 60 days, and 9 (47.3%) between 61 and 90 days. Several patients experienced readmissions across more than one interval, although none were readmitted in all three intervals.

In total, 8 patients (12.1% of the cohort) had exactly one readmission, while 11 patients (16.7%) had multiple readmissions. Specifically, 6 patients (9.1%) were readmitted twice, 4 patients (6.1%) were readmitted three times, and 1 patient (1.5%) was readmitted five times. Of these 11 patients with multiple readmissions, 6 patients experienced >1 readmissions due to reasons within the same category, including recurrent infection (for 2 patients with 2 readmissions and 1 patient with 3 readmissions) and persistent progressive disease (for 1 patient with 2 readmissions, 1 patient with 3 readmissions, and 1 patient with 5 readmissions).

### 3.2. Healthcare Utilization

#### 3.2.1. Initial Hospital Stay

**Figure 1:**
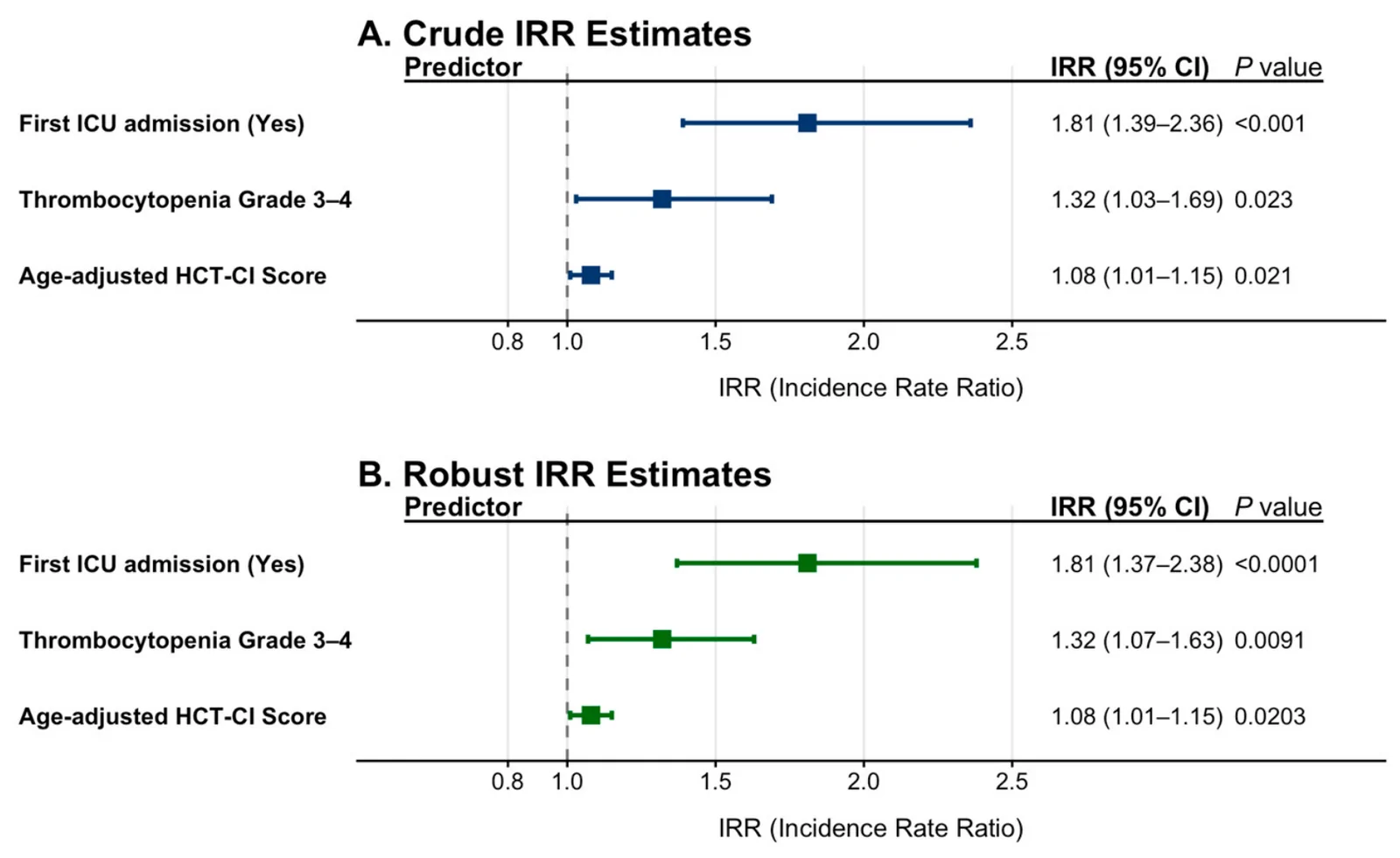
Risk Factors for Increased Initial LOS (Day 0 CAR-T Administration to Discharge). Multivariable negative binomial model with robust estimation. Model diagnostics indicated an appropriate variance structure (dispersion = 0.42), no significant multicollinearity (all VIFs < 2), and well-behaved residuals. Posterior predictive checks and residual uniformity plots confirmed good model fit. The final model (AIC = 439) outperformed the baseline negative binomial model (AIC = 478) and the Poisson model (AIC = 751).

The median initial hospital LOS was 12 days (range, 7–62); nineteen patients (29%) had an ICU admission during this period. Multivariable negative binomial model identified three significant predictors for increased LOS: age-adjusted HCT-CI score (IRR 1.08; 95% CI, 1.01–1.15; *p* = 0.0203), thrombocytopenia grade 3–4 (IRR 1.32; 95% CI, 1.07–1.63; *p* = 0.0091), and first ICU admission (IRR 1.81; 95% CI, 1.37–2.38; *p* < 0.0001) (Figure 1).

#### 3.2.2. 30-Day Readmission

Fourteen patients (21.2%) experienced an unplanned readmission within 30 days post-discharge. The median number of ER visits was 1 for those readmitted compared with 0 among non-readmitted patients (*p* < 0.001). The median LOS during readmission was 6 days (range, 0–35). There were no differences in other utilization parameters between readmitted and non-readmitted patients (Table 3). Notably, the ICU length of stay during initial hospitalization and number of outpatient visits up to 30 days were similar between the two groups.

We also evaluated post-infusion adverse events during patients’ initial hospital stay and subsequent 30-day hospital readmission (if applicable) following CAR-T therapy discharge (Table A1). There was a trend between infection severity and 30-day readmission (*p* = 0.069). A higher proportion of readmitted patients (50%) experienced grade 3–4 infection than non-readmitted patients (19%). Other adverse events (cytomegalovirus infection, ICANS, CRS) did not show statistically significant differences in relation to readmission status.

Multivariable logistic regression showed that grade 3–4 infection during initial hospitalization may be a signal for 30-day readmission (OR 12.1; 95% CI, 2.04–71.65; *p* = 0.0061) (Table A2). Similarly, multivariable negative binomial regression showed that longer initial hospital LOS also increased 30-day readmission LOS (IRR 1.23; 95%CI, 1.11–1.35; *p* = 0.0001). Outpatient follow-up visits showed a trend toward reduced odds of 30-day readmission (OR 0.51; 95% CI, 0.25–1.02; *p* = 0.0577), although this did not reach statistical significance (Table A2). In contrast, outpatient follow-up was significantly associated with shorter 30-day readmission LOS (IRR 0.43; 95% CI, 0.26–0.69; *p* = 0.0005) (Table A3).

#### 3.2.3. 90-Day Readmission

Nineteen patients (28.8%) were readmitted by Day 90 after initial hospital discharge. These patients had higher ER utilization, with a median of 2 ER visits at 90 days compared with 0 visits in non-readmitted patients. The median readmission LOS was 6 days (range, 0–35). Other utilization measures were generally comparable between groups, and outpatient visits showed an upward trend over time without significant group differences (Table 3).

Univariable logistic regression identified SUVmaxD30 as a significant predictor of 90-day rehospitalization (OR 1.09; 95% CI, 1.01–1.19; *p* = 0.031). Severe infection (grades 3–4) during the index hospitalization was also associated with an increased risk of rehospitalization (OR 8.00; 95% CI, 2.17–33.7; *p* = 0.013).

Within the multivariable negative binomial landmark analysis evaluating post-Day 30 readmission length of stay (Days 31–90), three variables remained independently associated with longer subsequent readmission LOS: higher Day 30 SUVmax (robust IRR 1.06; 95% CI, 1.03–1.09; *p* < 0.001), longer readmission LOS during the first 30 days following discharge (robust IRR 1.35; 95% CI, 1.14–1.60; *p* = 0.0006), and a greater cumulative number of emergency department visits within the first 60 days (robust IRR 3.10; 95% CI, 2.15–4.46; *p* < 0.001). In contrast, total initial hospitalization LOS was no longer significantly associated with subsequent readmission LOS after robust variance estimation (robust IRR 0.80; 95% CI, 0.59–1.07; *p* = 0.132) (Table 4).

#### 3.2.4. Multiple Readmissions

Among patients with exactly one readmission (*n* = 8), the median LOS was 2.5 days (range, 0–35). Meanwhile, patients with multiple readmissions (*n* = 11) had a median LOS of 6 days (range, 1–36) accounting for all hospitalizations.

Further stratification of the multiple readmission group revealed variability across subgroups. Patients with two (*n* = 6) or three readmissions (*n* = 4) had the longest stays, each with a median LOS of 8 days (range, 2–33 and 1–36, respectively). Those with two readmissions (*n* = 5) had a median LOS of 6.5 days (range, 2–33). The single patient with five readmissions had shorter stays overall, with a median LOS of 4 days (range, 3–14) (Table 5).

### 3.3. Impact of Healthcare Utilization on Survival Outcomes

To evaluate the long-term clinical implications of post-discharge healthcare utilization, overall survival was compared according to 90-day readmission status. Visual inspection of the Kaplan–Meier curves suggested modest crossing of the survival functions; therefore, both the conventional log-rank test and the Fleming–Harrington weighted log-rank test (ρ = 1, γ = 0) were performed. Neither test demonstrated a statistically significant difference in OS between patients who experienced at least one unplanned 90-day readmission and those who did not (log-rank *p* = 0.72; Fleming–Harrington weighted log-rank *p* = 1.00).

**Figure 2:**
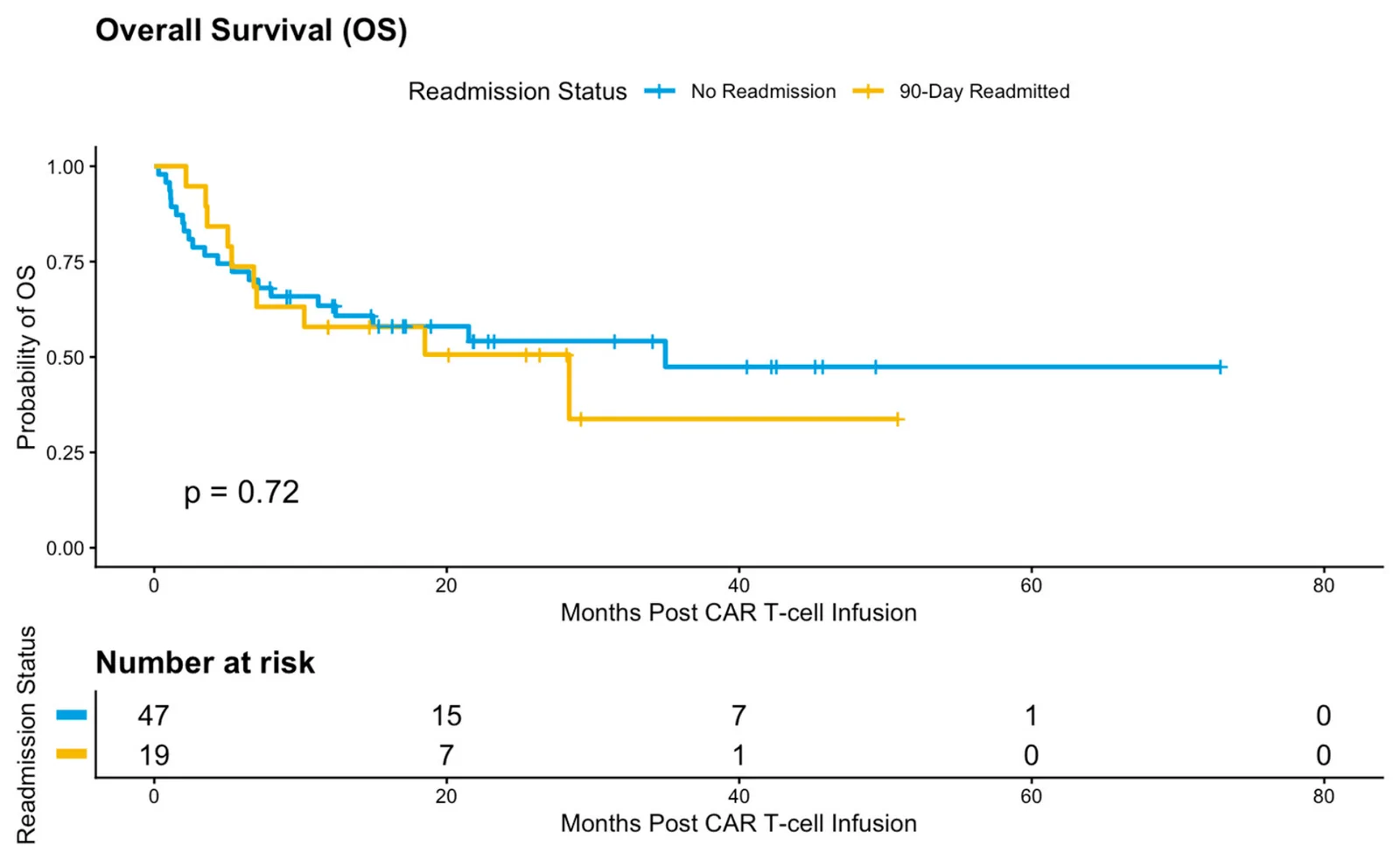
Overall Survival Comparison Between Patients With vs. Without 90-day Readmissions.

Median OS was 34.9 months (95% CI, 12.4 months to not reached) among patients without readmission and 28.4 months (95% CI, 7.0 months to not reached) among those with readmission (Figure 2).

## 4. Discussion

This retrospective cohort study provides a detailed evaluation of hospitalization patterns, including both the number and reasons for readmissions, as well as clinical predictors of utilization following CAR T-cell therapy for R/R LBCL in a real-world academic setting.

Unlike claims-based studies, we extracted clinical data manually, capturing information on inflammatory biomarkers (e.g., ferritin, CRP), ECOG status, CAR T-related toxicities, and imaging biomarkers such as SUVmax. This approach allowed us to examine clinically relevant predictors that are rarely included in cost or claims studies.

Notably, we identified that 19.7% of patients experienced readmissions across multiple timepoints (30-, 60-, and 90-days post-discharge). This sequential pattern has been largely overlooked in prior studies, which typically examine readmissions at single timepoints [9,12].

In this patient population, readmission rates at 30 days have been reported to range from 20% to 30% across settings and payer types [9,12,20]. We observed a 30-day readmission rate of 21.2% and 90-day readmission rate of 28.8%. Additionally, we found no differences in LOS or readmission outcomes by sex, which aligns with findings from larger cohorts [21,22]. Additionally, upon accounting for the likely evolution of CAR T-cell supportive care over time (with regard to toxicity management and discharge practices), when comparing 30/90-day readmission rates between 2016-2019 and 2020–2022 within our cohort, rates remained fairly similar: 23.1% vs. 20% at 30 days and 26.9% vs. 30% at 90 days.

Prior healthcare utilization reports in this patient population have demonstrated that inpatient CAR T-cell therapy administration is associated with high overall costs and a longer mean LOS of 17 days and an ICU admission rate of 20% [11,12,20]. Our findings of a median initial hospital LOS of 12 days and 29% ICU admission rate mirror prior studies. Our predictors aligned with a clinical intuition that the presence of a greater comorbidity burden (higher HCT-CI score), post-infusion complications (severe thrombocytopenia), and ICU admission would lead to an increased initial LOS. ICU LOS, especially, has been noted to play an important role within the CAR T-cell therapy-related LOS literature [23], which was consistent with our correlation assessment.

Post-infusion toxicities such as infection, CRS, and ICANS are well-documented complications of CAR T-cell therapy and are frequently implicated in rehospitalizations [6,11,13]. In our patient cohort, higher-grade infection (but not CRS or ICANS) was noted to be correlated with increased odds of 30-day readmission. Similar to the results of Sharma et al.’s study, the most common reasons for readmission within 30 days of discharge after CAR T-cell therapy in our cohort were infection (20%) and cancer progression (13.3%) [9]. The proportion with infection as the underlying etiology of readmission then increased to 37.8% within 90 days of CAR T-cell therapy discharge, aligning with the findings of large-scale meta-analyses that infections, rather than CAR T-cell-specific side effects (like CRS or ICANS), could be the most common cause of non-relapse mortality to be aware of in the CAR T-cell recipient population [24]. Specifically, neutropenic fever (without any identified organism), bacterial, and viral infections were the most common infectious etiologies of 90-day readmission in our cohort. With regard to infection prophylaxis, every patient in our cohort received herpes simplex virus/varicella zoster virus prophylaxis with acyclovir for one year after CAR T-cell therapy, Pneumocystis jirovecii pneumonia prophylaxis with sulfamethoxazole/trimethoprim (or an equivalent agent) for six months after CAR T-cell therapy, and fungal/bacterial prophylaxis with fluconazole/levofloxacin, respectively, for an absolute neutrophil count <500.

Generally speaking, compared to their counterparts, readmitted patients showed substantially greater ER utilization and higher cumulative readmission burden over 90 days, despite similar ICU usage and initial hospital LOS during inpatient CAR-T treatment. Consistent with the literature [8], these findings reinforce ER visits as an early warning signal of subsequent readmission.

Although patients who had unplanned 90-day readmission experienced substantially greater healthcare utilization, this increased utilization was not accompanied by significantly worse overall survival. These findings suggest that unplanned readmission primarily reflects greater clinical complexity and resource utilization rather than serving as an independent marker of adverse long-term prognosis. Alternatively, the absence of a detectable survival difference may reflect the effectiveness of intensive post-discharge monitoring and supportive care or may be attributable to the limited statistical power of this single-center cohort. Larger multicenter studies are needed to determine whether readmission burden independently influences long-term survival following CAR T-cell therapy.

Our findings also suggest a possible association between outpatient follow-up within the first 30 days after CAR T-cell therapy discharge and risk of 30-day readmission/duration of 30-day readmission LOS. However, the relationship between outpatient visit frequency and readmission risk is complex and should be interpreted with caution. Notably, patients who required closer outpatient monitoring may have had a higher baseline risk of complications, which could in turn increase their likelihood of readmission. This introduces the possibility of confounding by indication, whereby increased healthcare utilization reflects underlying clinical vulnerability rather than a protective effect of follow-up itself.

Beyond the initial 30-day period, the impact of outpatient follow-up appears less distinct. In our cohort, the median number of outpatient visits between readmitted and non-readmitted patients was similar at both 60 and 90 days (6 and 7 visits, respectively), suggesting that visit frequency alone may not adequately stratify risk in the later post-treatment period. Together, these findings highlight the need for more nuanced metrics of outpatient care, beyond visit frequency alone, to better understand its relationship with post–CAR T-cell outcomes.

Previous literature has revealed a link of Day 30 SUVmax ≥ 10 to disease progression risk [25,26] and high serum ferritin level as a potential biomarker for CRS/ICANS [27,28]. In our population with readmissions, ferritin and SUVmaxD30 appear elevated among readmitted patients, possibly indicating that unresolved CRS/ICANS and disease progression, respectively, may predispose individuals to recurrent admissions. The use of ferritin to predict underlying CRS/ICANS may be especially important to pay attention to during the early period after CAR T-cell therapy, as within the 30-day readmission group, CRS and/or ICANS were observed in 53.3% of all readmissions. This number then decreased to 18.9% within the 90-day readmission cohort. Thus, our results suggest that such biomarkers may help identify patients at higher risk for longer readmission LOS and escalating healthcare needs, which can be further stratified depending on the time interval from which they are post-CAR-T cell therapy.

Meanwhile, elevated SUVmaxD30 likely reflects persistent metabolically active lymphoma, early treatment resistance, or impending disease progression after CAR T-cell therapy. Prior studies have demonstrated that residual FDG avidity at Day 30 is associated with inferior progression-free and overall survival, suggesting that metabolic imaging may identify high-risk patients before overt clinical relapse [25]. In this context, patients with elevated SUVmaxD30 are at risk of greater healthcare utilization due to ongoing disease-related symptoms, progression-related complications, additional diagnostic evaluations, or the need for subsequent anti-lymphoma therapy. Furthermore, elevated metabolic activity following CAR T-cell therapy can reflect underlying biologic resistance mechanisms, including inadequate CAR T-cell expansion or persistence, T-cell exhaustion, antigen escape, or an immunosuppressive tumor microenvironment [29].

However, the use of SUVmax to prognosticate LBCL survival outcomes after CAR T-cell therapy has several drawbacks. These include the fact that SUVmax may not capture the full extent of disease burden (due to its emphasis on the most metabolically active foci in the body), it can easily be affected by technique and scanner variability, and it cannot clearly distinguish immune-related inflammation as a result of therapy (rather than true disease progression) [26]. As such, new imaging approaches, including dynamic PET and immunological positron emission tomography/single-photon emission computed tomography (immuno-PET/-SPECT), have emerged to try to address these challenges. Both methods rely on assessing the biodistribution of CAR T-cell products to more accurately detect resistance mechanisms and ongoing cytotoxicity after cellular therapy [29,30]. It is likely that the real-time monitoring advantages provided by these more advanced PET imaging techniques will be needed to better incorporate metabolic imaging into future prognostic models for CAR T-cell therapy response.

Another notable finding in this study was the burden of multiple readmissions. Nearly 17% of patients experienced two or more readmissions within 90 days. While most occurred within 30 days, a substantial proportion were also seen between 31–60 and 61–90 days, underscoring that complications extend beyond the traditional 30-day window used in quality metrics. Patients with two or three readmissions had the longest hospital stays, whereas the single patient with five readmissions had shorter but repeated admissions for management of progressive disease. These results highlight the need to consider both frequency and duration of hospitalization when evaluating healthcare burden. Moreover, infection (*n* = 3 patients) and progressive disease (*n* = 3 patients) were the most common recurring categorical etiologies for readmission, suggesting the presence of two distinct high-utilization phenotypes to be aware of among those with multiple readmissions, one characterized by repeated infections (possibly due to delayed immune recovery) and another represented by refractory disease.

Our findings further suggest that repeat hospitalizations cluster in predictable ways, and that early post-treatment indicators (e.g., ICU use and Day 30 SUVmax) may inform downstream healthcare utilization. Strong correlations between readmission metrics, ICU burden, and imaging biomarkers underscore the interconnected nature of post-CAR T-cell care needs.

Our study has several limitations. First, it was conducted at a single academic center, limiting generalizability. In particular, because 89% of patients received axi-cel, the findings primarily reflect an axi-cel-treated population and may not generalize to other CAR-T constructs. Second, our sample size (*n* = 66) restricts the power of subgroup analyses and may affect model stability; given the limited number of readmission events, multivariable analyses should be interpreted as exploratory and hypothesis-generating. To reduce overfitting, however, candidate variables were prespecified based on clinical relevance and prior literature before model selection. Third, we did not capture direct cost data or billing records, so our estimates of healthcare burden rely on LOS and service utilization rather than financial outcomes. Additionally, not all patients had clinical data (such as PET30/SUVmaxD30 results) available to be included in analyses; other important biologic variables (such as CAR T-cell expansion kinetics, inflammatory cytokine levels, frailty measures, etc.) were also unable to be assessed due to inadequate data availability. Finally, as a retrospective study, we cannot infer causality between predictors and outcomes, and residual confounding may remain. While collinearity was assessed using VIF, residual causal overlap remains possible between several variables that may represent different manifestations of the same clinical trajectory (e.g., ICU admission and hospital LOS).

Despite these limitations, our findings provide a more granular understanding of variables associated with healthcare utilization following CAR T-cell therapy. Our work may also guide future studies to further assess which pre-CAR T-cell treatment factors are associated with readmissions, thus helping inform strategies to optimize post-discharge care and guide approaches to risk-adapted post-discharge monitoring.

## 5. Conclusions

Our findings identify clinical and utilization factors associated with readmission burden following time of discharge after CAR T-cell therapy for R/R LBCL. Elevated SUVmax at Day 30, ICU admission, infection severity, and ER visits were associated with increased readmission risk or LOS. Conversely, outpatient follow-up within the first 30 days after discharge was associated with shorter readmission LOS, though this likely represents confounding by indication. These insights provide a foundation for targeted, risk-based post-discharge monitoring programs to reduce unplanned readmissions and improve resource allocation. Given that our study mainly offers hypothesis-generating associations, future multicenter studies with prospective interventions (e.g., early telehealth visits) are warranted to validate these predictors and improve outcomes for CAR T-cell therapy recipients.

## Figures and Tables

**Table 1:** Baseline Demographic and Clinical Characteristics by 30- and 90-day Readmission Status.

Characteristic	30-Day Readmission		90-Day Readmission	
	No (*n* = 52)	Yes (*n* = 14)	No (*n* = 47)	Yes (*n* = 19)
Male, *n* (%)	36 (69.2)	8 (57.1)	31 (66.0)	13 (68.4)
Age, years (SD)	58.35 (13.06)	56.71 (14.46)	58.09 (12.83)	57.79 (14.65)
Age ≥ 65 years, *n* (%)	19 (36.5)	5 (35.7)	17 (36.2)	7 (36.8)
Age ≥ 70 years, *n* (%)	11 (21.2)	4 (28.6)	9 (19.1)	6 (31.6)
Age-adjusted HCT-CI Score, median (range)	2 (0–6)	1.5 (0–8)	2 (0–6)	1 (0–8)
LDH Day 0, median (range)	265.5 (104–840)	276 (130–781)	282.5 (104–840)	225 (157–781)
CRP Day 0, median (range)	1.23 (0.05–18.43)	1.37 (0.3–16.53)	1.38 (0.05–18.43)	1.11 (0.27–16.53)
Ferritin Day 0, median (range)	776 (20–4661)	1759 (141–5830)	771 (51–4661)	1372.5 (20–5830)
Day 0 PET Deauville Score (PET0), mean (SD)	4.58 (0.78)	4.23 (1.48)	4.62 (0.77)	4.22 (1.31)
Day 30 PET Deauville Score (PET30), mean (SD)	N/A	N/A	3.50 (1.40)	3.46 (1.51)
Day 30 SUVmax, median (range)	N/A	N/A	3.35 (1.7–24)	9.4 (1.8–42.1)
ECOG Score 2–3, *n* (%)	11 (21.2)	2 (14.3)	11 (23.4)	2 (10.5)
Extranodal Involvement ≥ 2, *n* (%)	19 (36.5)	6 (42.9)	18 (38.3)	7 (36.8)
High-grade LBCL with gene rearrangements in MYC and BCL2, BCL6, or both, *n* (%)	26 (55.3)	8 (57.1)	24 (57.1)	10 (52.6)
Double Expressor, *n* (%)	13 (27.7)	4 (28.6)	11 (26.2)	6 (31.6)
Bridging Therapy, *n* (%)	29 (55.8)	8 (57.1)	26 (55.3)	11 (57.9)
Prior Lines of Therapy, median (range)	3 (1–7)	3.5 (1–6)	3 (1–7)	3 (1–5)
Prior Autologous Transplant, *n* (%)	13 (25.0)	6 (42.9)	12 (25.5)	7 (36.8)

Abbreviations: CRP, C-reactive protein (reference range, <0.5 mg/dL); ECOG, Eastern Cooperative Oncology Group; HCT-CI, Hematopoietic Cell Transplantation-specific Comorbidity Index; LBCL, large B-cell lymphoma; LDH, lactate dehydrogenase (reference range, 25–175 U/L); N/A, not applicable; PET, positron emission tomography; SUVmax, maximum standardized uptake value.

**Table 2:** Reasons for 30-Day and 90-Day Readmission After CAR T-cell Therapy Discharge.

Reason for Readmission	30-Day Readmission (*n* = 15 Readmissions)	90-Day Readmission (*n* = 37 Readmissions)
Infection, *n* (%)	3 (20.0)	14 (37.8)
Neutropenic fever of unclear source, *n* (%)	2 (13.3)	4 (10.8)
Disease Progression, *n* (%)	2 (13.3)	7 (18.9)
CRS, *n* (%)	2 (13.3)	2 (5.4)
ICANS, *n* (%)	4 (26.7)	4 (10.8)
Other, *n* (%)	4 (26.7)	10 (27.0)

**Table 3:** Healthcare Utilization Within 30 and 90 days of Discharge by Readmission Status.

	30-Day Readmission		90-Day Readmission	
Variables	No (*n* = 52)	Yes (*n* = 14)	No (*n* = 47)	Yes (*n* = 19)
Total Initial Hospital LOS (Day0 to Discharge), median days (range)	12 (7–62)	14 (7–55)	12 (7–62)	13 (7–55)
Total ICU LOS During Initial Hospitalization Mean days (SD) Median days (range)	2.35 (8.24) 0 (0–57)	2.79 (4.64) 0 (0–14)	2.57 (8.64) 0 (0–57)	2.11 (4.12) 0 (0–14)
Total number of ICU Admissions During Initial Hospitalization Mean (SD) Median (range)	0.38 (0.69) 0 (0–2)	0.57 (1.09) 0 (0–4)	0.40 (0.71) 0 (0–2)	0.47 (0.96) 0 (0–4)
30-Day Total Outpatient Visits, median (range)	4 (0–6)	3 (1–6)	3 (0–6)	4 (1–6)
60-Day Total Outpatient Visits, median (range)	N/A	N/A	6 (0–9)	6 (3–12)
90-Day Total Outpatient Visit, median (range)	N/A	N/A	7 (0–11)	7 (3–21)
30-Day ER Visit (yes/no), n_yes_ (%)	6 (11.5)	14 (100.0) *	3 (6.4)	17 (89.5)
60-Day ER Visit (yes/no), n_yes_ (%)	N/A	N/A	3 (6.4)	19 (100.0)
90-Day ER Visit (yes/no), n_yes_ (%)	N/A	N/A	3 (6.4)	19 (100.0)
30-Day ER Number of Visits, median (range)	0 (0–1)	1 (1–2) *	0 (0–1)	1 (0–2)
60-Day ER Number of Visits, median (range)	N/A	N/A	0 (0–1)	2 (1–4)
90-Day ER Number of Visits, median (range)	N/A	N/A	0 (0–1)	2 (1–6)
30-Day Readmission LOS, median days (range)	N/A	6 (0–35)	N/A	2.5 (0–35)
60-Day Readmission LOS, median days (range)	N/A	N/A	N/A	6 (0–35)
90-Day Readmission LOS, median days (range)	N/A	N/A	N/A	6 (0–35)

* *p* < 0.001.

**Table 4:** Predictors for Post-Day 30 (Days 31–90) Readmission LOS: Day 30 Landmark Analysis.

Predictors	Crude Estimates			Robust Estimates		
	IRR	95% CI	*p*-Value	IRR	95% CI	*p*-Value
Day 30 SUVmax	1.06	1.03–1.09	<0.001	1.06	1.03–1.09	<0.001
Total Initial Hospital LOS (Day0 to Discharge)	0.80	0.68–0.91	0.005	0.80	0.59–1.07	0.132
30-Day Readmission LOS	1.35	1.23–1.50	<0.001	1.35	1.14–1.60	0.0006
60-Day ER Number of Visits (Continuous)	3.10	2.13–4.68	<0.001	3.10	2.15–4.46	<0.001

Abbreviations: IRR = incidence rate ratio; CI = confidence interval. Negative binomial model with double robust estimation. Diagnostics indicated an appropriate variance structure (dispersion = 0.42), no significant multicollinearity (all VIFs < 4), and well-behaved residuals. Posterior predictive checks and residual uniformity plots confirmed good model fit. The final model (AIC = 93) outperformed both the baseline negative binomial model (AIC = 277) and the Poisson model (AIC = 1284). Outcome definition: The dependent variable represents readmission length of stay accumulated exclusively between Days 31 and 90 after discharge. Accordingly, all predictors precede the outcome period, consistent with a Day 30 landmark analysis.

**Table 5:** Readmission LOS in Patients with 1 Readmission and Multiple Readmissions.

Category	Patients, *n* (%)	Median LOS in Days (Range)
Exactly 1 readmission	8 (12.1)	2.5 (0–35)
>1 readmissions (all)	11 (16.7)	6 (1–36)
2 readmissions	6 (9.1)	8 (2–33)
3 readmissions	4 (6.1)	8 (1–36)
5 readmissions	1 (1.5)	4 (3–14)

## Data Availability

Research data that support the findings of this study are securely stored in an institutional repository and are available to share from the corresponding author upon reasonable request in compliance with the University of California San Diego’s Research Data Policy.

## Abbreviations

The following abbreviations are used in this manuscript:

AIC	Akaike Information Criterion
ALC	Absolute Lymphocyte Count
axi-cel	Axicabtagene Ciloleucel
CAR-T	Chimeric Antigen Receptor T-cell
CI	Confidence Interval
CMV	Cytomegalovirus
CRP	C-Reactive Protein
CRS	Cytokine Release Syndrome
CTCAE	Common Terminology Criteria for Adverse Events
DLBCL	Diffuse Large B-Cell Lymphoma
ECOG	Eastern Cooperative Oncology Group
ER	Emergency Room
HCT-CI	Hematopoietic Cell Transplantation–Comorbidity Index
ICANS	Immune Effector Cell-Associated Neurotoxicity Syndrome
ICU	Intensive Care Unit
IRR	Incidence Rate Ratio
LBCL	Large B-Cell Lymphoma
LDH	Lactate Dehydrogenase
LOS	Length of Stay
NB	Negative Binomial
OP	Outpatient
OR	Odds Ratio
PET0	Baseline PET Scan
PET30	Day 30 PET Scan
PR	Partial Response
R/R	Relapsed/Refractory
SD	Standard Deviation
SUVmaxD30	Maximum Standardized Uptake Value at Day 30
tisa-cel	Tisagenlecleucel
VIF	Variance Inflation Factor

## Appendix A

**Table A1:** Post-Infusion Safety Outcomes by 30-Day Readmission Status.

Adverse Events, *n* (%) *	No (*n* = 52)	Yes (*n* = 14)
Infection **, *n* (%)		
Grade 0	29 (55.8)	4 (28.6)
Grade 1–2	12 (23.1)	2 (14.3)
Grade 3–4	10 (19.2)	7 (50.0)
Grade 5	1 (1.9)	1 (7.1)
CMV Viremia, *n* (%)		
Grade 0	35 (67.3)	8 (57.1)
Grade 1–2	16 (30.8)	5 (35.7)
Grade 3–4	1 (1.9)	1 (7.1)
ICANS, *n* (%)		
Grade 0	25 (48.1)	4 (28.6)
Grade 1–2	15 (28.8)	5 (35.7)
Grade 3–4	12 (23.1)	5 (35.7)
CRS, *n* (%)		
Grade 0	7 (13.5)	1 (7.1)
Grade 1–2	44 (84.6)	13 (92.9)
Grade 5	1 (1.9)	0 (0.0)

* Based on Common Terminology Criteria for Adverse Events (CTCAE) [16]. ** CMV viremia excluded. Abbreviations: CMV, cytomegalovirus; CRS, cytokine release syndrome; ICANS, immune effector cell-associated neurotoxicity syndrome.

**Table A2:** Predictors for 30-Day Readmission: Crude and Robust Estimates.

Predictors	Crude Estimates			Robust Estimates		
	OR	95% CI	*p*-Value	OR	95% CI	*p*-Value
Infection						
Grade 1–2	1.91	0.22–14.5	0.5	1.91	0.29–12.38	0.4982
Grade 3–4	12.1	2.34–83.5	0.005	12.08	2.04–71.65	0.0061
CRS						
Grade 1–2	6.54	0.61–212	0.2	6.54	0.98–43.46	0.0521
30-Day total outpatient visits	0.51	0.25–0.94	0.041	0.51	0.25–1.02	0.0577

**Table A3:** Predictors for 30-Day Readmission LOS: Crude and Robust Estimates.

Predictors	Crude Estimates			Robust Estimates		
	IRR	95% CI	*p*-Value	IRR	95% CI	*p*-Value
CRP	0.90	0.72–1.14	0.400	0.90	0.71–1.14	0.386
Total initial hospital LOS (Day0 to Discharge)	1.23	1.00–1.62	0.029	1.23	1.11–1.35	0.0001
30-Day total outpatient visits	0.43	0.17–0.89	0.017	0.43	0.26–0.69	0.0005
